# European primary care research and a national general practice research agenda

**DOI:** 10.1080/13814788.2018.1559568

**Published:** 2019-02-22

**Authors:** Jelle Stoffers

**Affiliations:** Department of Family Medicine, Care and Public Health Research Institute (CAPHRI), Maastricht University, Maastricht, The Netherlands


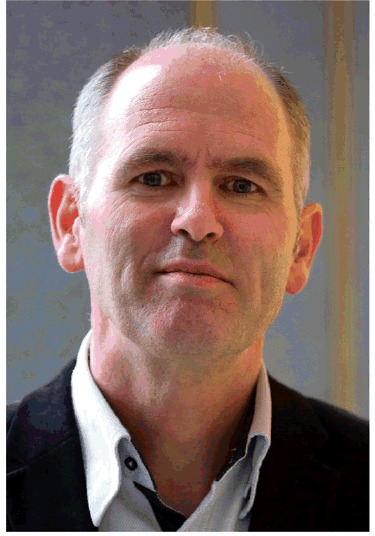
This Editor-in-Chief felt satisfied when he browsed the last volume of the *European Journal of General Practice* (EJGP). In 2018, we published 39 articles, including as many as 25 reports of original research. In addition to five articles with multinational authors, authors came from various countries across Europe, including Spain, France, Ireland, the UK, Belgium, the Netherlands, Latvia, Estonia, Germany, Poland, Switzerland, Hungary, Slovenia, Croatia, Serbia, and Turkey. The topics were as varied as a GP’s working day, reflecting many aspects of family medicine and primary care. Many papers were on clinical topics like cardiovascular diseases, mental health, gastrointestinal diseases, infections, or Parkinson’s disease. Furthermore, interdisciplinary collaboration (chronic disease management, oncology), palliative care and end-of-life medicine, quality and safety issues, including polypharmacy were important overarching themes. Healthcare reform was another relevant topic. We also published several papers on medical education. Our four-paper series on qualitative research methodology published in 2017 and 2018 apparently met a need: over 20 000 views altogether and 21 citations thus far [1,2]!

## Top-5 most valued articles

Of all these publications, like last year, the EJGP editors each picked three articles they found most valuable. The highest-ranking paper, mentioned by three editors, was the original research article by Katarina Vučićević and her colleagues from Serbia [3]. The paper tells us that safety issues are a key focus of patients when new medicines for long-term treatment are being prescribed to them. Adverse reactions are their main concern. During therapy initiation, around a quarter of patients indeed reported adverse drug reactions, and one-fifth of the interviewed patients experienced practical problems. Another paper favoured by three editors was the paper on antibiotic resistance by Albert Boada and his colleagues from Spain [4]. They found that penicillin-resistant staphylococci were statistically significantly associated with the number of packages of penicillin dispensed previously. Three other original research articles, each mentioned by one editor as his or her favourite publication, were about French junior lecturers in general practice, the consequences of health reform in Turkey, and how patients with Parkinson’s disease try to cope with changes in their care [5–7].

## Looking ahead to 2019 and beyond

As of now, we re-introduce the ‘four-issues-in-one-volume’ format. That way, we try to offer the visitors of the EJGP website a clear overview of our publications. In 2019, we hope to publish the first parts of a series on eHealth [8]. If you do research in this area yourself, I invite you to submit your work.

I want to highlight one publication in this first issue of Volume 25. I know an Editor-in-Chief should be reluctant in promoting articles written by colleagues from his home country. However, I should like to make an exception for the article on the Dutch research agenda for general practice [9]. The paper describes the methodology used to establish this research agenda and presents an example of research questions in the domain of ‘common diseases’. I encourage you to have a look for yourself. What do you think of the methodology? Would the results in your country have been similar? For those who are interested in the details a translated version of the research agenda is available at https://www.nhg.org/national-general-practice-research-agenda.

Finally, I wish you all have a good start in 2019 and I hope it will become a successful year with a lot to read, think, and write about.

Jelle Stoffers, Editor-in-Chief, the European Journal of General Practice,
*Department of Family Medicine*,
*Care and Public Health Research Institute (CAPHRI)*,
*Maastricht University*, *Maastricht*, *The Netherlands*,
ejgp-jstoffers@maastrichtuniversity.nl.

